# Comparison of Orthodontic Medicaid Funding in the United States 2006 to 2015

**DOI:** 10.3389/fpubh.2017.00221

**Published:** 2017-08-22

**Authors:** Gerald Minick, Terri Tilliss, W. Craig Shellhart, Sheldon M. Newman, Clifton M. Carey, Andrew Horne, Susan Whitt, Larry J. Oesterle

**Affiliations:** ^1^Department of Orthodontics, School of Dental Medicine, University of Colorado Anschutz Medical Campus, Aurora, CO, United States; ^2^Department of Restorative Dentistry, School of Dental Medicine, University of Colorado Anschutz Medical Campus, Aurora, CO, United States; ^3^Department of Craniofacial Biology, School of Dental Medicine, University of Colorado Anschutz Medical Campus, Aurora, CO, United States; ^4^University of Colorado Anschutz Medical Campus, Aurora, CO, United States

**Keywords:** medicaid database, orthodontic services, Medicaid dental expenditures, state expenditures, Medicaid funding, Medicaid reimbursement, Medicaid eligibility, affordable care act

## Abstract

**Introduction:**

Orthodontic treatment is reimbursed by Medicaid based on orthodontic and financial need with qualifiers determined by individual states. Changes in Medicaid-funded orthodontic treatment following the “Great Recession” in 2007 and the enactment of the Affordable Care Act in 2010 were compared for the 50 United States and the District of Columbia to better understand disparities in access to care. The results from this 2015 survey were compared to data gathered in 2006 (1).

**Materials and methods:**

Medicaid officials were contacted by email, telephone, or postal mail regarding the age limit for treatment, practitioner type who can determine eligibility and provide treatment, records required for case review, and rate and frequency of reimbursement. When not attained by direct contact, the information was gleaned from online websites, provider manuals, and state orthodontists.

**Results:**

Information gathered from 50 states and the District of Columbia documents that Medicaid program characteristics and expenditures continue to vary by state. Expenditures and reimbursement rates have decreased since 2006 and vary widely by geographic region. Some states have tightened restrictions on qualifiers and increased submission requirements by providers.

**Conclusion:**

The variation and lack of uniformity that still exists among Medicaid orthodontic programs in different states creates disparities in orthodontic care for US citizens. Barriers to care for Medicaid-funded orthodontic treatment have increased since 2006.

## Introduction

Medicaid funding for orthodontic services is a multifaceted issue with programmatic variation among states that can influence where orthodontists practice and who and how they treat. The Social Security Act was signed by President Lyndon Johnson in 1965. Title XIX of the Act, commonly known as Medicaid 1965 (2), was developed to provide healthcare coverage to the medically indigent. Title XIX listed certain medical services that states could fund with federal sharing. Orthodontics, although not specifically listed, was included with dental care (2). The Early and Periodic Screening, Diagnosis, and Treatment Program (EPSDT), established in 1967, is a component of Medicaid that provides preventive services and treatment for children and mandates access to orthodontic treatment for Medicaid eligible patients (3). *Handicapping malocclusions* were deemed eligible for Medicaid funding. With Medicaid financed half by the federal government and half by state government, it is at the discretion of individual states to define the term *handicapping malocclusion*. Consequently, there is wide disparity throughout the United States regarding Medicaid coverage of orthodontic treatment. There is a federal ceiling on income eligibility to limit expansion of the program beyond its original scope.

When Medicaid began in 1965, the American Dental Association (ADA) worked collaboratively with federal organizations to help define covered procedures and favored a national dental health program for children. A task force convened in 1966 recommended “treatment of malocclusion with priority provided for interceptive service and disfiguring or handicapping malocclusions” (2). Interceptive orthodontics, sometimes referred to as early orthodontics or Phase I treatment, has been shown to significantly reduce malocclusion severity in a comparison of Medicaid and private-pay populations (4). Improvements resulting from Phase I treatment can recategorize patients from the medically necessary category to the elective category, requiring less time and cost to treat (5, 6). However, such early orthodontic treatment may also improve a patients’ malocclusion enough to no longer have a *handicapping malocclusion* and thus be disqualified from receiving definitive orthodontic care. Consequently, the provision of Phase I treatment can present a conundrum regarding qualification for funding.

The American Association of Orthodontists (AAO) has defined medically necessary orthodontic care as “the treatment of a malocclusion (including craniofacial abnormalities/anomalies) that compromises the patient’s physical, emotional or dental health.” (7) The AAO originally selected the Salzmann index (8) as an objective qualifier for treatment funding for *handicapping malocclusions*. However, this decision was rescinded in 1985, with the AAO opposing the use of any index or classification system to determine orthodontic treatment need (9).

Since state budgets require funding decisions, most states still use an index as a qualifying criterion to define a *handicapping malocclusion*. Various malocclusion indices, sometimes with modifications, are used by states to serve their populations while meeting budget needs. With no standardization for determining qualified cases, disparity exists in orthodontic Medicaid case approvals. Moreover, states continue to alter criteria for funded care; the state of Iowa, for example, recently increased the case complexity required for approval, thus decreasing the number of cases funded per budget year (10). This raises the concern that patients in need are being disqualified from receiving treatment due to tightened state budgets.

Esthetic components of a malocclusion may or may not be considered by reviewers when determining cases to approve for funding. Some states use indices that include an esthetic component in addition to the study cast analysis. Examples of these indices are the Index of Complexity, Outcome and Need (11), Salzmann Index (8), Dental Aesthetic Index (12), and the Index of Treatment Need (13). Some states use indices that lack an esthetic component and rely purely on study cast analysis. These include the Handicapping Labiolingual Deviation (HLD) Index (14), Peer Assessment Rating Index (15), and the HLD (CalMod) Index (16). Use of study cast analysis only to determine treatment need may not give a clear picture of an existing visual deformity. Cast analysis alone frequently indicates that there is no need for orthodontic treatment; however, a visual assessment would have a different outcome.

Despite the EPSDT and Medicaid initiatives, which predicate federally required coverage, there are income, racial, ethnic, cultural, and geographic barriers limiting access to specialty dental care, including orthodontics. These and other barriers vary the rate of orthodontic care utilization by publicly insured children and adolescents. Disparities exist in the availability of orthodontic care for private versus publically insured youth in the United States (10, 17, 18). State to state variability in US orthodontic Medicaid programs also contributes to nation-wide geographical disparities.

Receiving state approval for funding of orthodontic treatment does not guarantee receipt of orthodontic care if an accessible care provider is not available. Medicaid reimbursement fees are substantially less than the usual and customary fees charged by dentists and orthodontists. Private practice office overhead has continued to increase since 2006. However, Medicaid reimbursement rates have decreased; in some states, the decrease is significant. In addition to lower reimbursement rates, Medicaid providers may have to hire additional staff to process the state required paperwork, submit required records, and follow-up on payments, thus increasing the office overhead. Consequently, some providers either choose not to accept Medicaid patients or severely restrict the number of Medicaid patients in their practice. As a result, individuals either go without care or are forced to travel, sometimes long distances, to obtain treatment.

To examine and compare the effects on Medicaid-funded orthodontic treatment that have occurred since the “Great Recession” starting in 2007 and the enactment of the Affordable Care Act (ACA) in 2010, a comparative study was designed to parallel the previously published study “Medicaid Expenditures for Orthodontic Services” (1).

## Materials and Methods

The methods and categories used in the 2006 study were repeated for comparison purposes. PubMed, Ovid, Google, Medicaid websites, and the state Medicaid Dental Services Section were accessed to identify the appropriate contact person for each of the 50 United States and District of Columbia. In addition, as much information as possible was gathered from state Medicaid websites. The identified person for each state was contacted by email and/or phone and secondarily by postal mail. For states where this person could not be ascertained or accessed, the information was acquired from that state’s general (non-dental) Medicaid office and orthodontist Medicaid providers. An introductory letter was sent by postal mail or email describing the 13-question multiple-choice survey, which, when necessary, was administered by phone. Data were analyzed with descriptive statistics and frequency distributions. Select tables replicate categories used in the 2006 study.

Although various forces in the external environment changed between 2006 and 2015, this study was a preliminary analysis to examine if these changes impacted Medicaid-funded orthodontic treatment. The study was not intended to analyze causation.

## Results

Email or postal mail responses were received by 43 states and the District of Columbia. For the remaining seven states (AZ, MA, RI, SC, SD, TN, and TX), as much information as possible was gathered from online websites, provider manuals, and published fee schedules. Patient websites were available for 33 states, and provider websites were available for 46 states. All states indicated provision of some services under Medicaid except for Michigan where orthodontic coverage is *via* another program for special needs beneficiaries with particular medical diagnoses such as cleft palate. This program is not under the auspices of Medicaid and utilizes a different funding source. Provider reimbursement rates for Michigan’s dental care program were included for comparison; however, since not participating specifically in Medicaid, other Michigan data were not included.

### Eligible Providers

**Figure 1:**
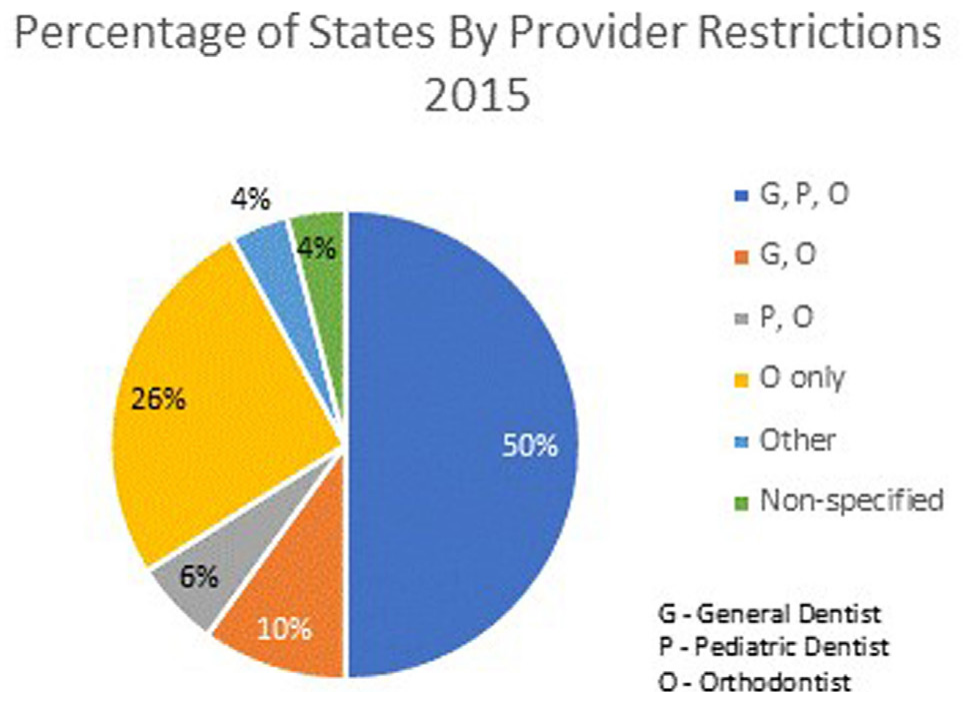
Percentage of states by provider restrictions 2015.

In 2015, 48 states specified the type of dentist eligible to provide Medicaid-funded orthodontic care. A general dentist, orthodontist, or pediatric dentist can provide such treatment in 25 states; orthodontists only in 13 states; either an orthodontist or a general dentist without restriction in 5 states; and only an orthodontist or pediatric dentist in 3 states. In Oklahoma, dentists are reimbursed through Medicaid for orthodontic services but must meet specific Oklahoma SoonerCare requirements. In Oregon, the provider can be any practitioner for whom the service is within the scope of practice. Arizona and Rhode Island did not specify eligible provider types (Figure 1).

Over the decade, some states have changed their rules regarding eligible providers. In 2006, 10 states restricted providers to be orthodontists. Since that time, six states (CO, IL, KS, MD, WV, and WY) changed to orthodontist only as a provider type, whereas three states moved away from restrictions to orthodontist only (DE, GA, and RI), allowing other dental practitioners to participate. Since the 2006 data did not include pediatric dentist as a category, a comparison could not be made.

### Coverage by Patient Age

**Figure 2:**
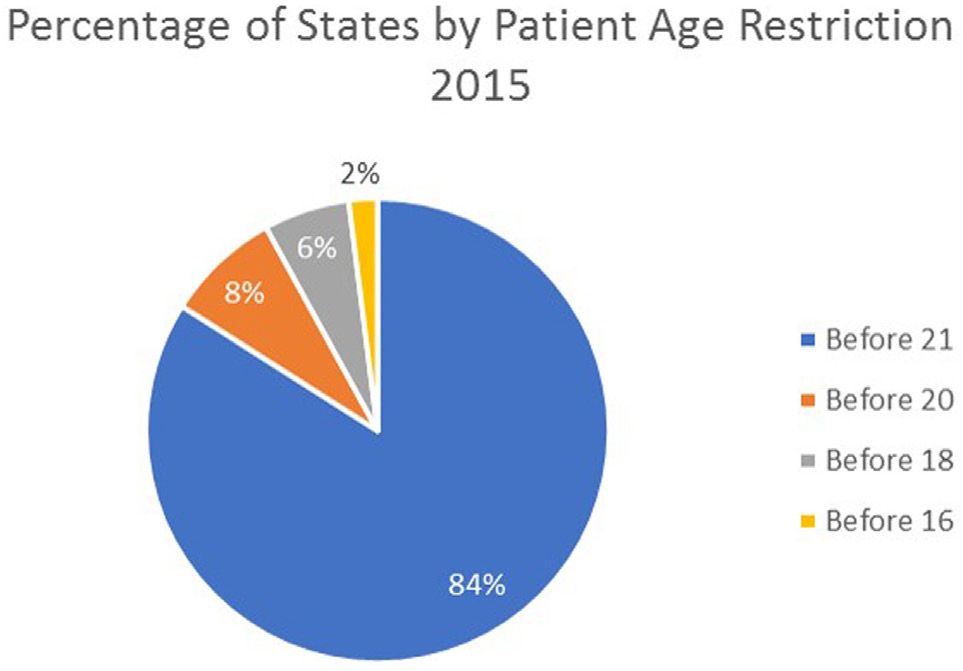
Percentage of states by age restriction 2015.

There are age limits for initiation of orthodontic treatment. In 2015, 42 states indicated that services must be initiated before age 21, before the age of 20 in 4 states, before age 18 in 3 states, and before the age of 16 in 1 state. Since 2006, 6 states have reduced the age for treatment initiation from before age 21 to before age 20 (NE, NV, TX, and UT) and before age 18 (NJ and OK). Oregon was the only state to increase the eligibility age by changing their restriction from age 18 to 21. Nine other states were listed in 2006 as “other” than 21. Six of those nine states previously listed as “other” have set the age for initiation of treatment before age 21 (AZ, CO, GA, LA, MN, and MT). The remaining three states (PA, SC, and WY) have specified eligibility ages as follows: PA, before age 23; SC, before age 16; and WY, before age 18 (Figure 2).

### Qualifying Criteria

Various indices are utilized to classify malocclusion in 41 states. In 2015, the HLD index was used by 15 states, the HLD Cal Mod index by 4 states, the Salzmann index by 4 states, and the Salzmann index plus additional criteria in 4 states. The PCP Statement of Medical Necessity, HLD (NJ Mod or RI Mod), Colorado Orthodontic Criteria Index form, Idaho Smiles Malocclusion Index, DentaQuest Orthodontic Criteria Index form, or a combination of these, is used by 14 states. The remaining nine states either do not use an index or failed to report its use.

In contrast, only 34 states reported using an index in 2006. The Salzmann index was the most common with 11 states utilizing it, followed by the HLD index (10 states). Other indices were reported being used in 13 states. The remaining 16 states either did not use an index or failed to report its use (Table 1).

**Table 1:** Comparison of the number of states using an index to determine qualification.

	2015	2006
HLD	15	10
Salzmann	4	11
Salzmann + Mod	4	-
HLD CA Mod	4	-
HLD RI Mod	1	-
HLD NJ Mod	1	-
ID Smiles	1	-
Other	11	13
Total	41	34

*HLD, Handicapping Labiolingual Deviation; Salzmann, Salzmann Index; Salzmann + MOD, Salzmann plus modifications; HLD CA Mod, Handicapping Labiolingual Deviation plus California Modifications; HLD Mod, Handicapping Labiolingual Deviation plus Rhode Island Modifications; HLD NJ Mod, Handicapping Labiolingual Deviation plus New Jersey Modifications; ID Smiles, Idaho Smiles Index*.

### Reviewers

The reviewer qualification for evaluating cases for eligibility varies by state. In 2015, the reviewer is exclusively an orthodontist in 18 states, must be a general dentist in 8 states, and exclusively a non-dentist in 6 states. Some states allow for more than one type of reviewer. For 11 states, the reviewer can be either an orthodontist or a general dentist. One state allows for an orthodontist or a non-dentist, whereas one other state allows for the reviewer to be an orthodontist, a general dentist, or a non-dentist. Five states did not report their criteria for reviewer qualification (AZ, KS, MA, SC, and TN) (Table 2).

**Table 2:** Comparison of the number of states utilizing specific reviewer types.

	2015	2006
O	18	26
O, G	11	6
G	8	12
ND	6	5
No response	5	1
O, ND	1	-
O, G, ND	1	-

*O, orthodontist; G, general dentist; ND, non-dentist*.

The number of reviewers required to approve cases varies by state. In 2015, 13 states required only a single reviewer while 32 states required more than 1 reviewer. Five states did not report the number of reviewers used for case approval. Comparisons were not available for 2006.

### Required Records

Records that must be submitted to assess eligibility vary by state and include combinations of models, cephalogram, panoramic radiograph, intraoral and extraoral photographs, tracings, treatment plans, PA cephalogram, signed statement from practitioner, and some additional forms. In 2015, study models were required in 27 states, cephalograms in 31 states, panoramic radiographs in 44 states, intraoral photos in 36 states, and other records were required in 29 states. By comparison, in 2006, study models were required in 31 states, cephalograms in 23 states, panoramic radiographs in 29 states, intraoral photos in 21 states, and other records in 29 states. Over the decade, more states are requiring submission of more types of records to justify Medicaid acceptance (Table 3).

**Table 3:** Comparison of the number of states requiring specific types of orthodontic records.

	2015	2006
Models	27	31
Cephalometric	31	23
Panoramic	44	29
Intraoral photos	36	21
Other	29	29
Total	167	133

### Reimbursement Methods to Providers

Reimbursement schedules varied in 2015 with 19 states reimbursing by a single payment, 2 states with annual payments, 6 states by quarterly payments, 7 states by monthly payments, and 8 states reporting “other” payment methods. Three states used a combination method of reimbursement and five states did not report their payment methods.

By comparison, in 2006, 12 states paid with a single payment, 1 state used annual payments, 1 state used biannual payments, 8 states paid quarterly, 13 states paid monthly, 14 states reported “other payment methods,” and for 1 state, there was no report (DC) (Table 4).

**Table 4:** Comparison of the number of states by reimbursement schedules.

	2015	2006
Single	19	12
Annual	2	1
Biannual	0	1
Quarterly	6	8
Monthly	7	13
Other	8	14
Combination	3	0
No response	5	1

Comparing the 2015 with 2006 reimbursement schedules, only 48% of states kept the same schedule for reimbursement, while 52% changed their reimbursement policy. The most prevalent change over time was a shift from quarterly or monthly reimbursement to a single payment.

### Acceptance Rates

Acceptance rates for submitted cases vary by state. Of the reporting states in 2015, 2 states had a 20–40% acceptance rate, 7 states had 40–60% acceptance, 6 states had 60–80% acceptance, and 14 states had an 80–100% acceptance rate. This information was not provided in the 2006 data.

### Expenditures

**Figure 3:**
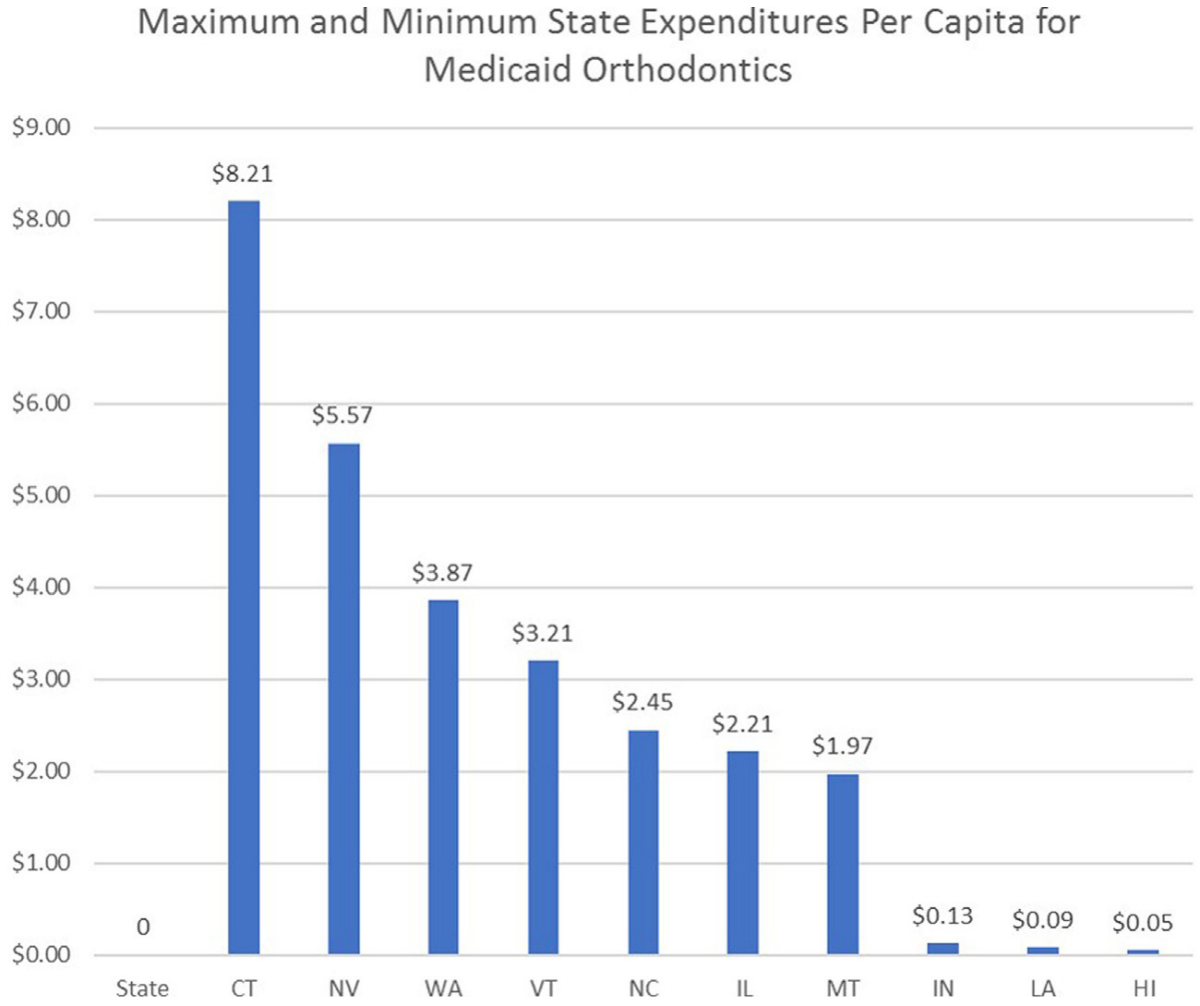
Highest and lowest state expenditures per capita for Medicaid orthodontics.

Total state expenditures varied from $75,242 to $29.5 million from FY 2013, 2014, or unspecified year. Total state expenditures were not reported in the 2006 data, so no comparisons were possible. The estimated cost of Medicaid orthodontic treatment per capita was calculated for select states by dividing the state orthodontic expenditures by the 2015 estimated state population data obtained from the US Census Bureau (Figure 3) (19).

### Reimbursement Rates

Reimbursement rates vary by state. For states with an initial payment followed by incremental payments based on treatment time, the reported rates are based on a 24-month comprehensive treatment time.

**Figure 4:**
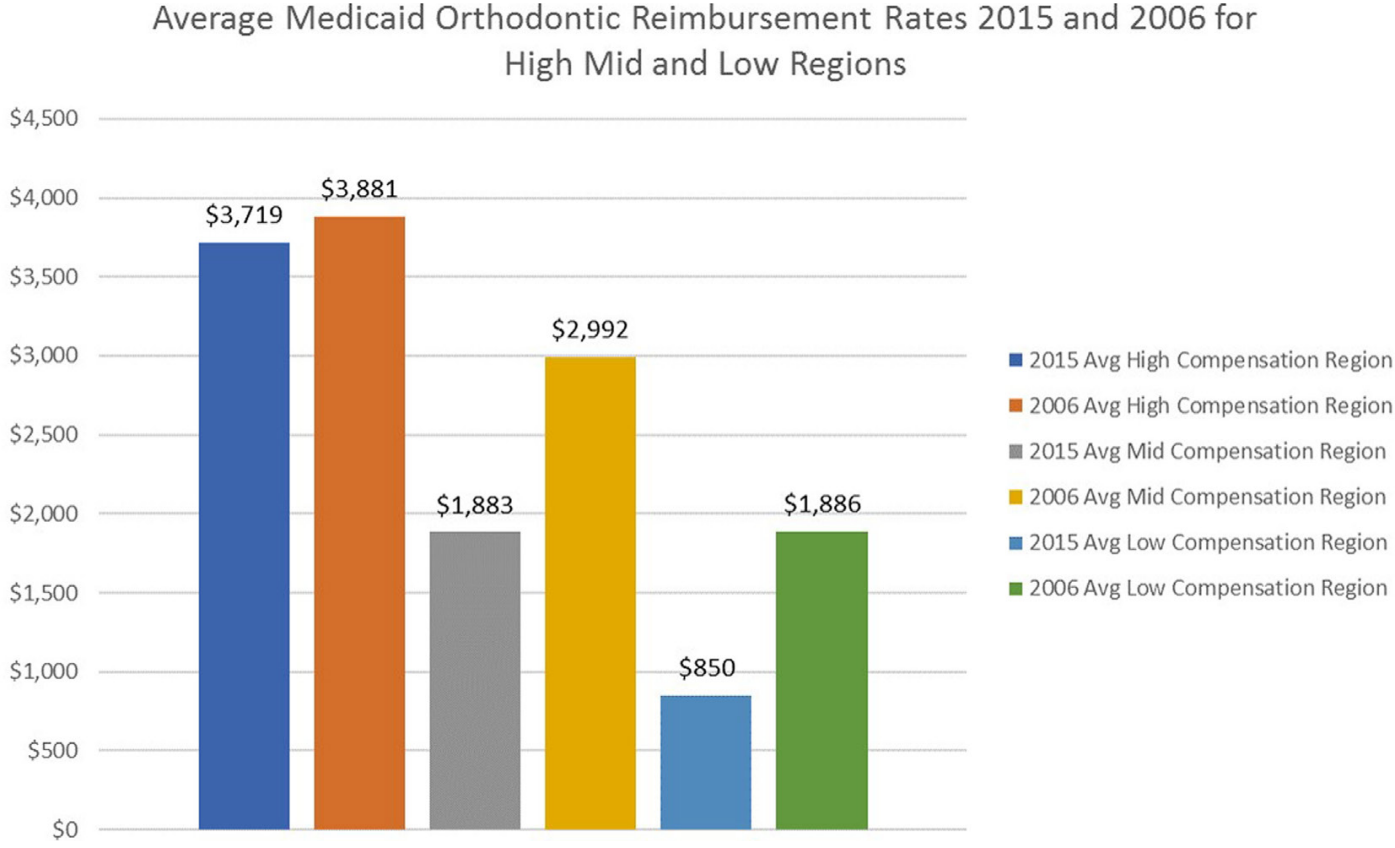
Comparison of Medicaid orthodontic reimbursement rates 2006 and 2015 for high, mid, and low regions.

States were grouped into highest, midrange, and lowest reimbursement rates to parallel the classification approach used in the 2007 publication (1). In 2015, the highest reimbursement group ranged from $2,847.43 to $5,044 per case with an average of $3,719 and a median of $3,600. The midrange group varied from $1,200 to $2,847.14 with an average of $1,883.46 and a median of $1,754.16. The lowest group ranged from $493 to $1,200 with an average of $850 and a median of $872.31 (Figure 4; Table 5). When compared to the 2006 data, all levels of reimbursement have decreased with the lowest reimbursement region experiencing the greatest percentage decrease.

**Table 5:** Medicaid orthodontic reimbursement rate change from 2006 to 2015 by region.

	2015			2006			% Change		
	Low	High	Average	Low	High	Average	Low	High	Average
Highest reimbursement region	$2,847	$5,044	$3,719	$3,200	$5,530	$3,881	−11.03%	−8.79%	−4.17%
Midrange reimbursement region	$1,200	$2,847	$1,883	$2,780	$3,178	$2,992	−56.83%	−10.41%	−37.05%
Lowest reimbursement region	$493	$1,200	$850	$775	$2,700	$1,886	−36.39%	−55.56%	−54.93%

Medicaid reimbursement rates were grouped by geographic region in the same manner as reported by El-Gheriani et al. to parallel reporting by the ADA (1, 20). For 2015, the regional averages were as follows: New England, $2,718; Middle Atlantic, $826; South Atlantic, $1,973; East South Central, $1,636; East North Central, $1,691; West North Central, $2,250; Mountain, $2,392; West South Central, $2,888; Pacific, $2,653. The overall average of the regions was $2,114 (Table 6).

**Table 6:** Comparison of 2006 and 2015 reimbursement averages by region.

Region	2015	2006	% Change
New England (CT, ME, MA, NH, RI, VT)	$2,719	$2,575	5%
Middle Atlantic (NJ, NY, PA)	$826	$2,336	−183%
South Atlantic (DE, DC, FL, GA, MD, NC, SC, VA, WV)	$1,973	$3,424	−74%
East South Central (AL, KY, MS, TN)	$1,636	$3,167	−94%
East North Central (IL, IN, MI, OH, WI)	$1,691	$3,226	−91%
West North Central (IA, KS, MN, NO, NE, ND, SD)	$2,250	$2,582	−15%
Mountain (AZ, CO, ID, MT, NV, NM, UT, WY)	$2,392	$3,162	−32%
West South Central (AR, LA, OK, TX)	$2,888	$2,801	3%
Pacific (AK, CA, HI, OR, WA)	$2,653	$3,225	−22%
Average	$2,114	$2,944	−39%

## Discussion

The financial crisis and resulting economic downturn that occurred in 2007 suggested the utility of updating the 2007 publication (1) to compare Medicaid expenditures for orthodontic services. Since the downturn, state budgets impacted by the nation’s economy have strategically reallocated available funds to meet fiscal needs. Reported reimbursement rates have decreased since data were collected in 2006. Due to federal mandate, dental and orthodontic coverage was not eliminated, but per case expenditures were reduced. The only regions for which reimbursement increased from 2006 to 2015 are the New England and West South Central regions, which when adjusted for inflation do not likely constitute an increase.

The gap between the economically advantaged and disadvantaged American communities has increased since the “Great Recession” ended and a slow economic recovery ensued (21). A 2016 study by the Economic Innovation Group (EIG) found that, while prosperous zip codes are more populous and have flourished during the recovery, the economically distressed zip codes continue to be exceptionally hard hit and have failed to participate in the economic recovery (22). Their findings suggest that a deep and ongoing recession continues in these areas of the country which is affecting 50.4 million Americans. During the period from 2010 to 2013, the most economically depressed areas continued to lose jobs at a rate of 13%. Instead of business growth occurring during this time period, 1 in 10 business establishments closed. This can be contrasted to the most economically prosperous areas of America that experienced a 22% employment rise and where business establishments increased by 11% (22).

By examining the country by zip code, EIG was able to determine the geographic location of many of the depressed regions. They found that most of the economically stressed areas are concentrated in the nation’s old industrial heartland and in the Deep South. By contrast, many of the prosperous areas are located in the Sun Belt and the western states. Areas such as the Rust Belt (Pennsylvania, West Virginia, Ohio, Indiana, Michigan, and Illinois) have experienced some economic rebound, but most of these states continue to languish in an economic recession (23).

In addition to the Great Recession, the ACA has impacted the healthcare system and state budgets since it was signed into law in 2010. Although the ACA was signed into law in 2010, changes in Medicaid did not take effect until January 1, 2014, with open enrollment beginning in October 2013. Under the new healthcare law, Medicaid, in general, underwent substantial changes including changes in eligibility and expanded coverage, modernization of the enrollment process, and increased outreach and enrollment efforts (24). The Kaiser Commission on Medicaid and the Uninsured found in a 2014 study that Medicaid and Children’s Health Insurance Program enrollment outpaced its usual rate by an additional 4.8 million people (8.2% increase) within the first 6 months after the new Medicaid rules of the ACA went into effect (24).

While the ACA has been successful at reducing the number of uninsured Americans, it has also strained state budgets by rapidly increasing the number of Medicaid recipients receiving state-funded medical coverage (24). Since state budgets are funded by tax dollars that are collected from economic activity occurring within a state, economically stressed states have felt a disproportionate amount of the financial burden of the ACA.

If a direct comparison between 2006 and 2015 of the number of dentists providing Medicaid orthodontic treatment were possible, it seems likely that the 2015 data would show a greater disparity in access to care among income groups due to changes in Medicaid eligibility, availability of providers, and a host of other factors; however, this is difficult to accurately measure. The authors of a recent study suggest that it is difficult to determine how many dentists actually participate in Medicaid due to the uncertainty created by indirect measurement techniques, since provider participation rates are often estimated by how extensively providers bill Medicaid and treat beneficiaries. Estimated low rates of dentist participation have often resulted in expressed criticism of dentistry for not sufficiently serving Medicaid beneficiaries (25).

Orthodontic care, while it is important and part of the federal mandate, may not be considered as critical as other medical procedures. As a result, since 2006, it is likely that states have reallocated some of their resources and reduced expenditures for orthodontics to reduce pressure on strained medical budgets. Since the 2006 study did not include state expenditures for orthodontic care, a direct comparison was not possible. However, in 2015, the importance some states have placed on provision of orthodontic care was illustrated by the state expenditures per capita in those states. On the upper end of the spectrum, Connecticut and Nevada spent $8.21 and $5.57, respectively, per capita. On the lower end of the spectrum, Louisiana and Hawaii spent on $0.09 and $0.05, respectively, per capita for provision of orthodontic care (Figure 3).

By comparing the highest, midrange, and lowest reimbursement groupings from the 2006 study to 2015, it is apparent that provider reimbursement has decreased (Figure 4). The greatest reduction of reimbursement over the past decade is in the middle and low reimbursement regions. The national average for Medicaid orthodontic reimbursement to providers decreased by 28% from 2006 to 2015 (Table 7). Regional comparisons of average Medicaid reimbursement rates generally reveal a decrease over the last decade, even without applying an inflation adjustment (Table 6). Some regions have seen larger decreases than others. Comparing the East North Central Region (comprised largely by Rust Belt states that have not shared as much in the economic recovery) to the Pacific Region, it is apparent that reimbursement rates have decreased significantly more in the East North Central Region (Table 8).

**Table 7:** Comparison of adolescent orthodontic treatment reimbursement rates 2006 and 2015.

	2015	2006	% Change
Average Medicaid reimbursement	$2,114	$2,944	−28%
Average private practice reimbursement	$5,194	$4,670	11%
Medicaid as% of private practice reimbursement	41%	63%	−35%

**Table 8:** Comparison of reimbursement rates between private practice fees and Medicaid fees for the East North Central Division and the Pacific Division 2006–2015 (20).

	2015	2006	% Change
**East North Central Region (IL, IN, MI, OH, WI)**			
Average Medicaid reimbursement	$1,691	$3,226	−48%
Average private practice reimbursement	$5,229	$4,660	12%
Medicaid as% of private practice reimbursement	32%	69%	−53%
**Pacific Region (AK, CA, HI, OR, WA)**			
Average Medicaid reimbursement	$2,653	$3,225	−18%
Average private practice reimbursement	$5,354	$4,889	10%
Medicaid as % of private practice reimbursement	50%	66%	−25%

Comparison of private practice fees versus public reimbursement reported by the ADA 2016 Survey of Dental Fees (20) by selected region reveals that the discrepancy between private versus public pay has widened substantially (Table 8). Even economically stressed areas have seen increases in private practice orthodontic reimbursement rates from 2006 to 2015.

In addition to decreasing Medicaid reimbursements, the reluctance of some orthodontists to treat Medicaid patients relates to the fact that Medicaid funding can cease if a patient is no longer Medicaid qualified, even though orthodontic treatment is incomplete. This has the most impact in states that utilize a periodic reimbursement schedule. Orthodontists may be unwilling to treat a large number of Medicaid patients for fear of continued treatment needs long after payment for orthodontic services has been discontinued.

It is possible that, if existing laws were rewritten so that Medicaid was solely a federally subsidized program without state-based variability, equal access to care would improve. This would require the federal government to set reimbursement rates for regions using an approach similar to that utilized by private insurance companies. As long as reimbursement rates were kept reasonably competitive, compared to local fees, orthodontists would be encouraged to treat Medicaid patients, improving access for those currently underserved.

Restriction of types of dentists permitted to provide orthodontic care from 2006 to 2015 has decreased the number of Medicaid providers in several states. Eight states (CO, FL, IL, KS, MD, TN, WV, and WY) that allowed general dentists to provide orthodontic care in 2006 have restricted care to specialists in 2015. Medicaid-funded orthodontics can be provided only by an orthodontist in six states (CO, IL, KS, MD, WV, and WY). Pediatric dentists and orthodontists are permitted to provide care in two states (FL and TN). Since qualification for orthodontic care is limited to *handicapping malocclusions*, Medicaid-funded orthodontic cases by definition are more complex and often more difficult to treat successfully. While some may reasonably argue that specialists are better equipped to provide orthodontic care to these individuals, states that restrict care to specialists make it more difficult for patients to identify local providers. The Kaiser Family Foundation reports that as of April 2017, there were 146,526 actively practicing general dentists, 6,093 pediatric dentists, and 6,147 orthodontists in the United States. In the 6 states that have restricted Medicaid-funded orthodontics to orthodontists only, there are 14,778 general dentists, 541 pediatric dentists, and 882 orthodontists in active practice. Since orthodontists only constitute 6% of dentists licensed to and likely to perform orthodontic treatment in theses states, it is possible that patients may be forced to seek orthodontic treatment a distance from their community, adding an access to care barrier (26).

Patient age limits for treatment initiation have lowered in some states. While most states stipulate that orthodontic treatment must begin prior to a patient’s 21st birthday, six states have reduced the age limit since 2006. The restriction is age 20 for four states (NE, NV, TX, and UT) and age 18 for two states (NJ and OK). The state of Oregon was the only state to raise the age restriction from 18 to 21 years during that time period. By lowering the age requirement, states decrease the number of potential patients that can be approved for Medicaid-funded orthodontic treatment. However, patients with the most severe malocclusions often require a combination of orthodontics and orthognathic surgery to achieve a successful result. In many cases, orthognathic surgery should only be performed once growth is complete, which for males is often in their early 20s (27). If states decrease the age limitation too severely, people with severe malocclusions most in need of corrective orthodontic treatment may be excluded, benefiting state budgets but not individuals.

Another mechanism for reducing the number of funded cases is the increased use of malocclusion indices. The number of states using indices to determine eligibility increased from 34 in 2006 to 41 in 2015. Although more objective, some indices do not consider the esthetic component of an individual’s malocclusion. By removing a reviewer’s ability to approve cases that constitute an obvious *handicapping malocclusion*, but fail to score appropriately on an index, states deny care to patients who are in need of orthodontic treatment. Some forms of *handicapping malocclusion* are not readily apparent without the use of human intelligence.

States have increased the number and types of records that must be provided by a practitioner to determine case eligibility (Table 3). The number of states requiring lateral cephalograms, panoramic radiographs, and intraoral photos has increased since 2006. In theory, the use of these records should increase the ability of the state reviewer to determine the need for treatment. However, it also increases patient chair time and overhead costs. For example, in 2015, reimbursement for intraoral photos ranged from $59 to $0 with an average reimbursement of $14. However, 19 states that require intraoral photographs for treatment approval do not reimburse for them. If the submission requirements become too arduous, providers may decide the additional hassle, and cost associated with provision of Medicaid orthodontic care is not justified. As a result, their acceptance of Medicaid patients will either be reduced or discontinued in favor of privately insured or fee-for-service patients, further increasing disparities.

In summary, decreases in Medicaid funding and changes in regulations and practices across states have resulted in considerable difference in the access to orthodontic care for handicapping malocclusions. The reasons for these changes are primarily economic but result in barriers of access for those in need. Further research could be done to examine policies and practices that could be altered to improve access.

### Limitations

(1) The study spans the time frame of the Great Recession and the passage and rollout of the ACA, but the study is not designed to analyze causation. (2) The methodology follows that used in the 2006 study. Not all data collected in 2015 were gathered in 2006, making some comparisons impossible. Furthermore, the data categories collected in the 2006 study, such as geographic areas and reimbursement levels, were repeated in 2015 to allow comparisons. Other categories may have been selected for the 2015 study if direct comparisons had not been the goal. (3) The study does not include data on patients or providers, both of which might add information to considerations of barriers to (or disparities in) access to care.

## Conclusion

1. There is extensive variation among Medicaid-funded orthodontic programs in the United States.
2. In the past decade, reimbursement rates for orthodontic services generally decreased by a range of 115–283%.
3. Continued regional economic strain and increased Medicaid enrollment resulting from the enactment of the ACA may be responsible for reductions in Medicaid-funded orthodontic reimbursements and tighter qualifiers for case acceptance.
4. Differences between state Medicaid programs create disparities in orthodontic care depending on a citizen’s state of residency.

## Declarations

Neither the institution nor any authors received payment or services from a third party for any aspect of submitted work. There are no financial relationships that have influenced or given the appearance of potentially influencing this work. There are no patents, copyrights, or royalties relevant to this work. There are no relationships or activities that have influenced or perceived to influence the content of this work.

## Author Contributions

GM and TT have written the re-submission. WS, SN, CC, AH, SW, and LO have all been involved in the project.

## Conflict of Interest Statement

The authors declare that the research was conducted in the absence of any commercial or financial relationships that could be construed as a potential conflict of interest. The reviewer, PB, and the handling editor declared their shared affiliation, and the handling editor states that the process nevertheless met the standards of a fair and objective review.
